# Prognostic value of preoperative MELD-albumin score in patients undergoing heart transplant

**DOI:** 10.3389/fcvm.2025.1675714

**Published:** 2026-01-12

**Authors:** Zhou Wei, Liu Xiao-bin, Xu Zhi-yun, Han Lin, Zhu Feng, Li Bai-ling

**Affiliations:** 1 Department of Cardiovascular Surgery, The First Affiliated Hospital of Naval Medical University (Shanghai Changhai Hospital), Shanghai, China; 2 Department of Critical Care Medicine, Shanghai East Hospital, Tongji University School of Medicine, Shanghai, China

**Keywords:** association, heart transplantation, MELD-Albumin score, model for end-stage liver disease score, prognosis

## Abstract

**Objective:**

Heart transplantation (HT) is the ultimate treatment option for patients with end-stage heart failure, and its prognostic evaluation has consistently been a focal point in clinical research. This article primarily explores the impact of the pre-operative Model for End-Stage Liver Disease (MELD) and its derivative scoring systems on the prognosis of HT patients.

**Methods:**

A retrospective analysis was conducted on the data of patients who underwent HT at Shanghai Changhai Hospital from January 2018 to January 2024. All included patients were scored using the MELD and its upgraded versions (MELD-XI, MELD-albumin). Initially, the preoperative baseline of survival group and non-survival group were compared. Subsequently, the association between various MELD scores and patient prognosis was analyzed using the Receiver Operating Characteristic (ROC) curve. Based on the higher Area Under the Curve (AUC), MELD-albumin was selected as the research indicator. Patients were then divided into high-score group and low-score group according to its optimal cutoff value, and the perioperative data of the two groups were compared.

**Results:**

A total of 170 patients were included in this study, with 159 patients (93.5%) in survival group and 11 patients (6.5%) in non-survival group. Comparison of preoperative and intraoperative baseline data between the two groups revealed that the non-survival group had a lower preoperative platelet count, higher preoperative creatinine levels and BNP levels, a lower left ventricular ejection fraction (LVEF), and higher scores in MELD, MELD-XI, and MELD-albumin compared to the survival group. ROC analysis demonstrated that the AUC values for preoperative MELD scores in predicting in-hospital mortality were 0.806, 0.842 for MELD-XI, and 0.843 for MELD-albumin. MELD-albumin was selected as the primary indicator. Based on the optimal cutoff value of 8.4, patients were divided into low-score group (MELD-albumin ≤8.4, 128 cases) and high-score group (MELD-albumin >8.4, 42 cases) to explore its relationship with perioperative prognosis in HT. The results showed statistical differences between the two groups in preoperative white blood cell count, platelet count, monocyte count, bilirubin, creatinine, BNP, international normalized ratio (INR), and procalcitonin levels, while no statistical differences were observed in intraoperative data. Regarding prognosis, the high-score group had a higher mortality rate (19% vs. 2.3%, *P* = 0.0004) and a higher proportion of patients suffering postoperative acute kidney injury (AKI) (38% vs. 18.7%, *P* = 0.023) and receiving continuous renal replacement therapy (CRRT) (14.3% vs. 4.7%, *P* = 0.039).

**Conclusion:**

This study confirms that the preoperative MELD-albumin score is an independent predictor of in-hospital mortality in HT patients, and its optimal cutoff value of 8.4 can effectively distinguish between high-risk and low-risk populations.

## 1 Introduction

Heart transplantation (HT) serves as the ultimate therapeutic option for patients with end-stage heart failure (1), and its prognostic evaluation has consistently been a focal point of clinical research. The Model for End-Stage Liver Disease (MELD) primarily encompasses objective laboratory parameters, including the international normalized ratio (INR), serum bilirubin, and creatinine (2). Currently, it has emerged as the primary scoring tool for prioritizing patients awaiting liver transplantation (3). In recent years, the application of MELD and its derivative scoring systems in the field of cardiovascular surgery has gradually garnered attention (4, 5). Among them, MELD-Albumin (a modified model that replaces INR with albumin) has become an important predictive tool due to its ability to integrate hepatic and renal function with nutritional status. The traditional MELD model evaluates hepatic and renal function through three indicators: bilirubin, creatinine, and INR. However, INR is susceptible to interference from non-hepatic disease factors, leading to potential scoring biases. MELD-Albumin innovatively substitutes serum albumin for INR, considering that hypoalbuminemia is a significant marker of liver dysfunction, malnutrition, and acute-phase responses. By incorporating albumin, the new scoring model can better predict short-term mortality in liver transplantation patients (6). This article primarily explores the impact of the preoperative MELD-Albumin scoring model on the prognosis of HT patients.

## 2 Materials and methods

### 2.1 Study subjects

A retrospective analysis was conducted on the data of patients who underwent HT at department of Cardiovascular surgery, Shanghai Changhai Hospital, The Naval Medical University from January 2018 to January 2024.

### 2.2 Study methods

For all selected study subjects, variable data were cleaned, encoded, and preprocessed to ensure data accuracy and completeness. Patients were then scored using the MELD and its upgraded versions (MELD-XI, MELD-albumin). Initially, based on the patients’ perioperative prognosis (28-day mortality), they were divided into survival group and non-survival group. The preoperative baseline data of the two groups were compared, with particular attention to the correlation between various MELD scores and prognosis. Subsequently, ROC analysis was performed to assess the association between these MELD scores and patient prognosis. Given the higher AUC values, MELD-albumin was selected as the research indicator. Patients were grouped into high-score group and low-score group based on its optimal cutoff value, and the perioperative data of the two groups were compared.

### 2.3 Calculation formulas for study indicators

MELD = 3.78 × ln(total bilirubin) + 11.2 × ln(INR) + 9.57 × ln(serum creatinine) + 6.43 × (etiological correction factor);
MELD-XI = 5.11 × ln(total bilirubin) + 11.76 × ln(serum creatinine);
MELD-albumin = 3.78 × ln(total bilirubin) + 9.57 × ln(serum creatinine) + 5.1 × ln(albumin) + 6.43 × (etiological correction factor).

### 2.4 Informed consent and ethical statement

This study is a retrospective observational case-control study that does not involve active intervention or additional data collection from subjects. All patients provided informed consent and signed informed consent forms. The study was approved by the Ethics Committee of the First Affiliated Hospital of Naval Medical University after review, with the approval number: CHEC2025-398.

### 2.5 Statistical analysis

Statistical analysis was performed using SPSS 22.0 software. For measurement data that conformed to a normal distribution and met the assumption of homogeneity of variance, they were expressed as mean ± standard deviation (x ± s), and comparisons between two groups were made using independent samples *t*-tests. Otherwise, data were expressed as median (lower quartile, upper quartile), and comparisons between two groups were made using non-parametric rank-sum tests. Categorical data were expressed as counts and percentages, and comparisons between two groups were made using the *χ*^2^ test. *P*-value < 0.05 was considered statistically significant.

## 3 Results

A total of 170 patients were included in this study, with 159 patients (93.5%) in the survival group and 11 patients (6.5%) in the non-survival group. Comparison of preoperative and intraoperative baseline data between the two groups revealed that the non-survival group had a lower preoperative platelet count [140.00 (83.00, 187.00) vs. 171.00 (136.00, 207.00), *P* = 0.033], higher preoperative creatinine levels [1.93 (1.24, 3.63) vs. 0.97 (0.69, 1.21), *P* < 0.001], higher preoperative BNP levels [4,663.53 (1,275.91, 5,000.00) vs. 1,275.91 (638.51, 2,852.98), *P* = 0.007], lower LVEF [19.00 (15.00, 22.00) vs. 24.00 (20.00, 32.00), *P* = 0.006], and higher scores in MELD [11.29 (8.15, 18.39) vs. 7.74 (5.48, 9.25), *P* < 0.001], MELD-XI [14.26 (12.02, 16.15) vs. 9.83 (7.77, 11.41), *P* < 0.001], and MELD-albumin [10.48 (8.43, 11.62) vs. 6.68 (5.01, 7.98), *P* < 0.001]. No statistically significant differences were observed in intraoperative conditions (Table 1).

**Table 1:** Patient characteristics by in-hospital death.

	In-hospital death			
Variable	Overall *N* = 170^a^	Survival *N* = 159^a^	Non-survival *N* = 11^a^	*p*-value^b^
Preoperative data				
Gender (Male, %)	132 (78%)	122 (77%)	10 (91%)	0.46
Age	52.00 (39.00, 60.00)	52.00 (38.00, 59.00)	52.00 (43.00, 63.00)	0.67
Cardiomyopathy	137 (81%)	129 (81%)	8 (73%)	0.45
T2DM	23 (14%)	22 (14%)	1 (9.1%)	>0.99
Hypertension	41 (24%)	39 (25%)	2 (18%)	>0.99
Coronary artery disease	22 (13%)	19 (12%)	3 (27%)	0.16
Heart valve disease	50 (29%)	47 (30%)	3 (27%)	>0.99
COPD	3 (1.8%)	3 (1.9%)	0 (0%)	>0.99
WBC count (10^9^ /L)	7.67 (5.91, 9.97)	7.57 (5.81, 9.88)	8.33 (7.46, 18.35)	0.11
Platelet count (10^9^ /L)	171.00 (133.00, 207.00)	171.00 (136.00, 207.00)	140.00 (83.00, 187.00)	0.033
Monocyte count (10^9^ /L)	0.69 (0.52, 1.09)	0.68 (0.50, 1.09)	0.88 (0.57, 4.60)	0.37
Lymphocyte count (10^9^ /L)	1.73 (1.09, 2.80)	1.75 (1.12, 2.80)	1.10 (0.48, 2.84)	0.085
Bilirubin (mg/dL))	1.41 (0.87, 2.05)	1.35 (0.87, 2.05)	1.71 (1.22, 3.62)	0.11
Serum creatinine(mg/dL)	0.98 (0.71, 1.27)	0.97 (0.69, 1.21)	1.93 (1.24, 3.63)	<0.001
AST (U/L)	26.00 (19.00, 52.00)	26.00 (19.00, 48.00)	24.00 (21.00, 275.00)	0.37
ALT (U/L)	25.00 (19.00, 50.00)	25.00 (19.00, 50.00)	32.00 (13.00, 189.00)	0.90
Albumin (g/L)	40.00 (37.00, 43.00)	40.00 (37.00, 44.00)	38.00 (34.00, 41.00)	0.16
BNP (ng/L)	1,275.91 (701.00, 3,038.00)	1,275.91 (638.51, 2,852.98)	4,663.53 (1,275.91, 5,000.00)	0.007
INR	1.17 (1.05, 1.40)	1.17 (1.05, 1.38)	1.40 (1.16, 2.04)	0.071
PCT (ng/mL)	0.58 (0.58, 0.59)	0.58 (0.58, 0.58)	0.58 (0.58, 1.97)	0.13
LVEF (%)	24.00 (19.00, 31.00)	24.00 (20.00, 32.00)	19.00 (15.00, 22.00)	0.006
mPAP (mmHg)	24.00 (21.00, 29.00)	24.00 (21.00, 28.00)	31.00 (24.00, 36.00)	0.11
MELD	7.81 (5.65, 9.53)	7.74 (5.48, 9.25)	11.29 (8.15, 18.39)	<0.001
MELD-XI	9.96 (7.85, 12.02)	9.83 (7.77, 11.41)	14.26 (12.02, 16.15)	<0.001
MELD-albumin	6.79 (5.14, 8.39)	6.68 (5.01, 7.98)	10.48 (8.43, 11.62)	<0.001
Operative data				
CPB time (min)	149.00 (120.00, 173.00)	148.00 (117.00, 172.00)	168.00 (125.00, 181.00)	0.17
Aortic cross clamp time (min)	45.00 (38.00, 53.00)	45.00 (38.00, 53.00)	45.00 (40.00, 56.00)	0.35
Ischemia time (min)	188.00 (72.00, 348.00)	190.00 (78.00, 350.00)	132.00 (63.00, 315.00)	0.25
Assist circulation time (min)	95.00 (58.00, 115.00)	95.00 (58.00, 113.00)	99.00 (57.00, 121.00)	0.70

T2DM, type 2 diabetes mellitus; COPD, chronic obstructive pulmonary disease; WBC, white blood cell; AST, aspartate aminotransferase; ALT, alanine aminotransferase; BNP, B-type natriuretic peptide; INR, international normalized ratio; PCT, Procalcitonin; LVEF, left ventricular ejection fraction; PAP, pulmonary artery pressure; CPB, cardiopulmonary bypass; MELD, model for end-stage liver disease.

a Median (Q1, Q3) or Frequency (%).

b Fisher's exact test; Wilcoxon rank sum test.

**Figure 1:**
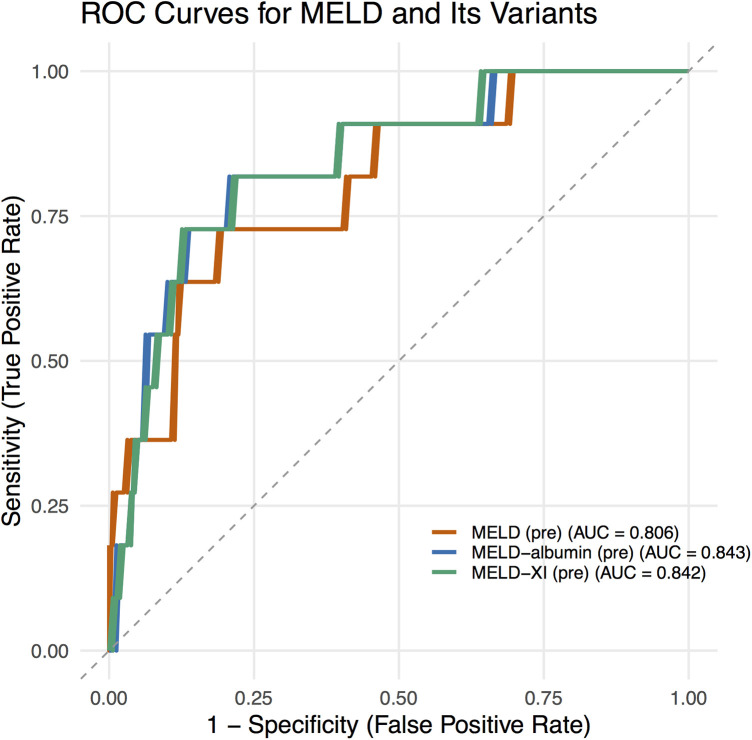
ROC curves for MELD scores.

ROC analysis demonstrated that the AUC values for preoperative MELD scores in predicting in-hospital mortality were 0.806 for MELD, 0.842 for MELD-XI, and 0.843 for MELD-albumin (Figure 1), all indicating good predictive value.

**Figure 2:**
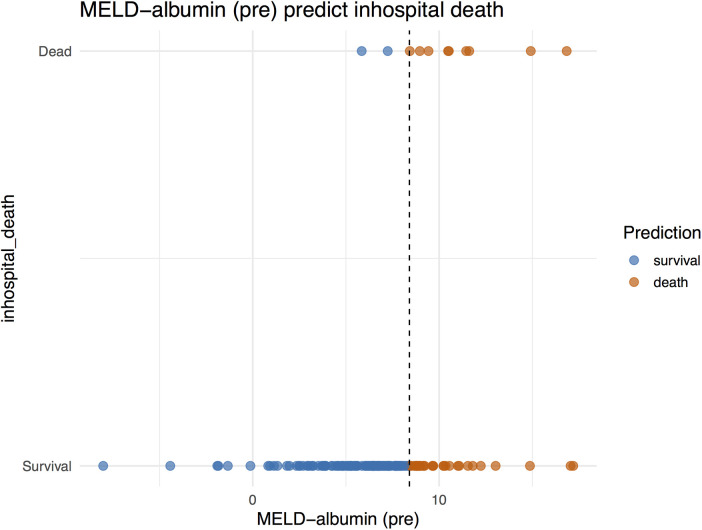
MELD−albumin predict in-hospital death.

Decision analysis plots were simultaneously generated to illustrate the ability of the MELD−albumin (pre) scoring system to predict the risk of in-hospital mortality. These plots visually presented the predictive effect of MELD−albumin (pre) on patient prognosis. As the score increased, the patients’ risk of mortality also rose. At lower scores, the prediction line approached the survival zone, indicating a lower risk of mortality for patients; conversely, as scores increased, the prediction line approached the mortality zone, indicating a higher risk of mortality for patients (Figure 2).

Based on the superior AUC, MELD-albumin was selected as the primary indicator. Patients were divided into low-score group (MELD-albumin ≤8.4, 128 patients) and high-score group (MELD-albumin >8.4, 42 patients) according to the optimal cutoff value of 8.4, to explore the relationship between MELD-albumin and perioperative prognosis in HT. The results showed statistically significant differences between the two groups in preoperative white blood cell count, preoperative platelet count, preoperative monocyte count, preoperative bilirubin, preoperative creatinine, preoperative BNP, preoperative INR, and preoperative procalcitonin levels. No statistically significant differences were observed in intraoperative data. In terms of prognosis, the high-score group had a higher mortality rate (19% vs. 2.3%, *P* = 0.0004) and a higher proportion of patients suffering postoperative acute kidney injury (AKI) (38% vs. 18.7%, *P* = 0.023) and receiving continuous renal replacement therapy (CRRT) (14.3% vs. 4.7%, *P* = 0.039) (Table 2).

**Table 2:** Patient characteristics by MELD–albumin (pre) group.

	MELD–albumin (pre) group			
Variable	Overall *N* = 170^a^	Low group (≤8.4) *N* = 1,28^a^	High group (>8.4) *N* = 42^a^	*p*-value^b^
Preoperative data				
Gender (Male, %)	132 (78%)	95 (74%)	37 (88%)	0.061
Age	52.00 (39.00, 60.00)	52.50 (38.50, 59.00)	52.00 (39.00, 62.00)	0.66
Cardiomyopathy	137 (81%)	104 (81%)	33 (79%)	0.70
T2DM	23 (14%)	19 (15%)	4 (9.5%)	0.38
Hypertension	41 (24%)	32 (25%)	9 (21%)	0.64
Coronary artery disease	22 (13%)	15 (12%)	7 (17%)	0.41
Heart valve disease	50 (29%)	37 (29%)	13 (31%)	0.80
COPD	3 (1.8%)	3 (2.3%)	0 (0%)	>0.99
WBC count (10^9^ /L)	7.67 (5.91, 9.97)	7.35 (5.68, 9.66)	8.45 (7.46, 10.90)	0.004
Platelet count (10^9^ /L)	171.00 (133.00, 207.00)	180.50 (144.50, 210.00)	143.50 (108.00, 181.00)	0.001
Monocyte count (10^9^ /L)	0.69 (0.52, 1.09)	0.67 (0.49, 0.96)	0.85 (0.60, 4.50)	0.025
Lymphocyte count(10^9^ /L)	1.73 (1.09, 2.80)	1.74 (1.17, 2.82)	1.70 (0.60, 2.45)	0.30
Bilirubin (mg/dL))	1.41 (0.87, 2.05)	1.16 (0.84, 1.77)	2.37 (1.85, 3.63)	<0.001
Serum creatinine(mg/dL))	0.98 (0.71, 1.27)	0.87 (0.57, 1.05)	1.49 (1.27, 2.48)	<0.001
AST (U/L)	26.00 (19.00, 52.00)	26.00 (19.00, 41.00)	24.00 (18.00, 104.00)	0.45
ALT (U/L)	25.00 (19.00, 50.00)	24.00 (19.00, 46.00)	31.00 (19.00, 69.00)	0.23
Albumin (g/L)	40.00 (37.00, 43.00)	40.00 (36.00, 43.50)	39.00 (37.00, 43.00)	0.52
BNP (ng/L)	1,275.91 (701.00, 3,038.00)	1,156.75 (549.34, 2,656.28)	1,895.00 (1,060.12, 4,712.00)	0.002
INR	1.17 (1.05, 1.40)	1.14 (1.04, 1.31)	1.31 (1.17, 1.60)	0.002
PCT (ng/mL)	0.58 (0.58, 0.59)	0.58 (0.35, 0.58)	0.58 (0.58, 1.31)	0.018
LVEF (%)	24.00 (19.00, 31.00)	24.00 (19.50, 31.00)	24.50 (19.00, 29.00)	0.85
mPAP (mmHg)	24.00 (21.00, 29.00)	24.00 (20.00, 26.00)	24.00 (24.00, 31.00)	0.049
Operative data				
CPB time (min)	149.00 (120.00, 173.00)	150.00 (120.50, 172.00)	140.50 (110.00, 180.00)	0.75
Aortic cross clamp time (min)	45.00 (38.00, 53.00)	45.00 (38.00, 52.50)	45.00 (40.00, 58.00)	0.35
Ischemia time (min)	188.00 (72.00, 348.00)	251.50 (104.00, 350.00)	106.00 (63.00, 338.00)	0.055
Assist circulation time (min)	95.00 (58.00, 115.00)	97.00 (62.50, 115.50)	83.00 (48.00, 113.00)	0.11
Postoperative data				
Mechanical assist	36 (21%)	26 (20%)	10 (24%)	0.63
IABP (*n*, %)	24 (14%)	18 (14%)	6 (14%)	0.82
ECMO (*n*, %)	12 (7.1%)	8 (6%)	4 (5%)	0.51
ARF (*n*, %)	22 (12.9%)	14 (10.9%)	8 (19%)	0.17
AKI (*n*, %)	40 (23.5%)	24 (18.7%)	16 (38%)	0.023
CRRT (*n*, %)	12 (5.9%)	6 (4.7%)	6 (14.3%)	0.039
Mortality (*n*, %)	11(6.5%)	3(2.3%)	8(19%)	0.0004

T2DM, type 2 diabetes mellitus; COPD, chronic obstructive pulmonary disease; WBC, white blood cell; AST, aspartate aminotransferase; ALT, alanine aminotransferase; BNP, B-type natriuretic peptide; INR, international normalized ratio; PCT, procalcitonin; LVEF, left ventricular ejection fraction; PAP, pulmonary artery pressure; CPB, cardiopulmonary bypass; MELD, model for end-stage liver disease; IABP, intra-aortic balloon pump; ECMO, extracorporeal membrane oxygenation; ARF, acute respiratory failure; AKI, acute kidney injury; CRRT, continuous renal replacement therapy.

a Median (Q1, Q3) or Frequency (%).

b Pearson's Chi-squared test; Fisher's exact test; Wilcoxon rank sum test.

## 4 Discussion

For the preoperative assessment of HT recipients, previous efforts have primarily focused on evaluating patients’ cardiopulmonary function, utilizing methods such as cardiopulmonary exercise testing (7, 8) and invasive hemodynamic monitoring via right heart catheterization. These approaches aim to assess the severity of heart failure, guide optimized medical treatment, and evaluate the presence of pulmonary hypertension to assess its impact on post-transplant right heart failure and survival (9–11). Additionally, various prognostic scoring systems have been developed for risk stratification in patients with heart failure (12). The Heart Failure Survival Score (HFSS), Seattle Heart Failure Model (SHFM), Meta-Analysis Global Group in Chronic Heart Failure (MAGGIC) score, and Metabolic Exercise Test Data combined with Cardiac and Kidney Indexes (MECKI) score were specifically developed for patients with chronic heart failure (13–15). It should be noted that all scoring systems have inherent limitations, with most performing poorly when applied to individual patients (as opposed to populations), particularly due to the lack of assessment models for organ function in HT recipients. In this study, we innovatively applied the MELD-Albumin scoring model to HT recipients to validate its effectiveness as a preoperative assessment tool.

To our knowledge, MELD score originally developed for sequence of liver assessment and liver transplantation, with the progress of heart-related research helping to diagnose more subtle heart abnormalities, especially those overlooked in patients with liver cirrhosis, an increasing amount of data shows that heart dysfunction precedes the prediction of liver and kidney function development in patients (16). Patients with heart failure are prone to malnutrition and blood dilution, which may aggravate their liver function (17). Clinically, non-invasive imaging examinations such as abdominal ultrasound showing signs of liver congestion (dilation of the superior hepatic vein and inferior vena cava) can provide support (18). Rapid and transient increases in other laboratory tests such as aminotransferase and lactate dehydrogenase levels are typical manifestations of liver function impairment (19, 20). In particular, a serum ALT/LDH ratio less than 1.5 in the early stage is a characteristic of cardiogenic injury (21). Our findings revealed that patients with a preoperative MELD-Albumin score ≥8.4 had a significantly higher in-hospital mortality rate and required a higher proportion of CRRT for organ support postoperatively. These results confirm the critical role of the MELD-Albumin score in predicting postoperative outcomes in HT.

Notably, patients in the non-survival group exhibited lower preoperative platelet counts, elevated creatinine and BNP levels, and reduced LVEF. These findings suggest that renal dysfunction, poorer preoperative cardiac systolic function, and coagulation disorders are high-risk factors for mortality in HT patients. During preoperative assessment, it is crucial to place sufficient emphasis on evaluating organ function. The MELD-Albumin score, by integrating creatinine (reflecting renal function), bilirubin (reflecting hepatic function), and albumin (reflecting nutritional status and inflammatory response), comprehensively quantifies the degree of multi-organ dysfunction in patients, thereby enabling accurate prediction of postoperative mortality risk.

In our study, ROC analysis demonstrated that the AUC value of MELD-Albumin reached 0.843, outperforming both MELD (0.806) and MELD-XI (0.842). This indicates that the integration of albumin into the scoring system further enhances its prognostic predictive capability. Albumin is not only a core component in maintaining plasma colloid osmotic pressure but also possesses multiple biological functions, including antioxidant, anti-inflammatory, and endotoxin-binding properties. Hypoalbuminemia can arise from various causes, such as liver damage during the acute phase of inflammatory processes, increased renal excretion, malnutrition, heightened catabolism, intestinal losses, severe volume overload, and escape into the interstitial space (22). It serves as a robust, reliable, and dependent prognostic indicator in patients with conditions including coronary artery disease (CAD) and heart failure (23), and is particularly prevalent among those with end-stage heart failure. A retrospective review of 1,726 patients with heart failure and reduced ejection fraction (HFrEF) (aged 52 ± 13 years, LVEF 23 ± 7%) revealed that 25% of patients had hypoalbuminemia (≤3.4 g/dL), with a 1-year survival rate of 66% among those with hypoalbuminemia compared to 83% in those without (*p* < 0.0001) (24). Serum albumin is a potent predictor of death or heart failure hospitalization (25) and is an easily overlooked prognostic indicator (26). Given its reliable predictive value, serum albumin is routinely tested in all hospitalized patients. Its inclusion in the MELD score significantly enhances its prognostic value, especially in the preoperative assessment of heart failure recipients awaiting HT.

The optimal cutoff value for MELD-Albumin in predicting the prognosis of HT patients identified in this study was 8.4, which slightly differs from previously reported optimal cutoff values in other cardiovascular diseases. For instance, a study evaluating 152 patients undergoing isolated tricuspid valve replacement (ITVR) found that the MELD-albumin score was an independent predictor of in-hospital mortality, with an optimal cutoff value of 8.58 determined by maximally selected log-rank statistics (27). This discrepancy may stem from several factors: First, differences in population characteristics: patients included in this study may have comprised more HT recipients without a cirrhosis background (e.g., those with dilated cardiomyopathy or ischemic cardiomyopathy), who exhibited more severe liver and kidney dysfunction. Second, limitations in sample size: the relatively small sample size in this study may have led to estimation bias in the cutoff value. Future research should involve multi-center, large-sample studies to establish a unified cutoff value, thereby enhancing the generalizability of the MELD-Albumin score.

## 5 Limitations

Our study also has several limitations. As a single-center retrospective design, due to the limited sample size, it may be susceptible to selection bias and information bias. Additionally, the lack of long-term follow-up data on 1-year or 5-year survival rates and the analysis of only 28-day mortality rates may impose limits the analysis to in-hospital mortality. Finally, we did not incorporate biomarkers such as inflammatory factors (e.g., IL-6) or myocardial injury markers (e.g., TnI), which may have omitted critical prognostic information.

## 6 Conclusion

Our study confirms that the preoperative MELD-Albumin score serves as an independent predictor of in-hospital mortality in HT patients. With an optimal cutoff value of 8.4, it effectively distinguishes between high-risk and low-risk populations, thereby demonstrating its potential as a valuable tool for preoperative organ function assessment in HT recipients.

## Data Availability

The raw data supporting the conclusions of this article will be made available by the authors, without undue reservation.
